# Parliament and the Professions

**Published:** 1919-11-22

**Authors:** 


### PARLIAMENT AND THE PROFESSIONS.

### Ministry of Health Reports.

SIR C. KINLOCH-COOKE asked the Minister of Health why a report in a recent outbreak of small-pox had not been furnished to the Isle of Wight County Council. Dr. Addison said that the report made by the medical inspector was a confidential document intended solely for the information of the Ministry, and he saw no sufficient reason in the present case to warrant a departure from the established practice in the case of such reports.

### Tuberculous Ex-Service Men.

DR. ADDISON stated that a scheme for the immediate provision at existing sanatoria of facilities for the training of tuberculosis ex-Service men in suitable occupations has been prepared by the Ministry of Health, and he hoped to make an announcement on the subject in a few days.

### Demobilisation of Medical Practitioners.

MAJOR FARQUHARSON asked the Secretary of State for War as to the total number of medical practitioners who have been demobilised since April 1, 1919, and as to how many holding temporary commissions are still detained in the East. Mr. Churchill stated that 4,823 had been demobilised since the date mentioned, and that those at present serving are as follows: Mesopotamia, 173; India, 152; Egypt, 244; these totals including a small number (which was detailed in the reply) in respect of whom a special recommendation for early release was received from the Ministry of National Service prior to April 1919. A large number of officers are at present embarking for the United Kingdom from the countries in question.

### Mentally Deranged Ex-Soldiers.

IN answer to a question, the Minister of Pensions stated that mentally deranged ex-soldiers who have been removed to Rainhill Pauper Lunatic Asylum are not treated as pauper lunatics but as private patients, the cost of their maintenance and of the special privileges which private patients enjoy being borne by the Ministry of Pensions.

